# Poly[[diaqua­bis­{μ-4-[6-(4-carb­oxy­phen­yl)-4,4′-bipyridin-2-yl]benzoato-κ^2^ *O*:*N* ^1′^}copper(II)] dimethyl­formamide tetra­solvate]

**DOI:** 10.1107/S1600536813006430

**Published:** 2013-03-13

**Authors:** Yabin Sun, E Song, Daguang Wang

**Affiliations:** a Department of Ophthalmology, The First Hospital of Jilin University, Changchun, 130021, People’s Republic of China; b Department of Gastrointestinal Surgery, The First Hospital of Jilin University, Changchun, 130021, People’s Republic of China

## Abstract

In the title compound, {[Cu(C_24_H_15_N_2_O_4_)_2_(H_2_O)_2_]·4C_3_H_7_NO}_*n*_, the Cu^II^ ion, lying on an inversion center, is six-coordinated by two N atoms from two 4-[6-(4-carb­oxy­phen­yl)-4,4′-bipyridin-2-yl]benzoate (*L*) ligands, two deprotonated carboxyl­ate O atoms from two other symmetry-related *L* ligands and two water mol­ecules in a slightly distorted octa­hedral geometry. The Cu^II^ atoms are linked by the bridging ligands into a layer parallel to (101). The presence of intra­layer O—H⋯O hydrogen bonds and π–π inter­actions between the pyridine and benzene rings [centroid–centroid distances = 3.808 (2) and 3.927 (2) Å] stabilizes the layer. Further O—H⋯O hydrogen bonds link the layers and the dimethyl­formamide solvent mol­ecules.

## Related literature

For the design of metal-organic coordination polymers, see: Ge & Song (2012 ▶); Herm *et al.* (2011 ▶); Liu *et al.* (2010 ▶); Wang *et al.* (2010 ▶). For a related structure, see: Xia *et al.* (2012 ▶). 
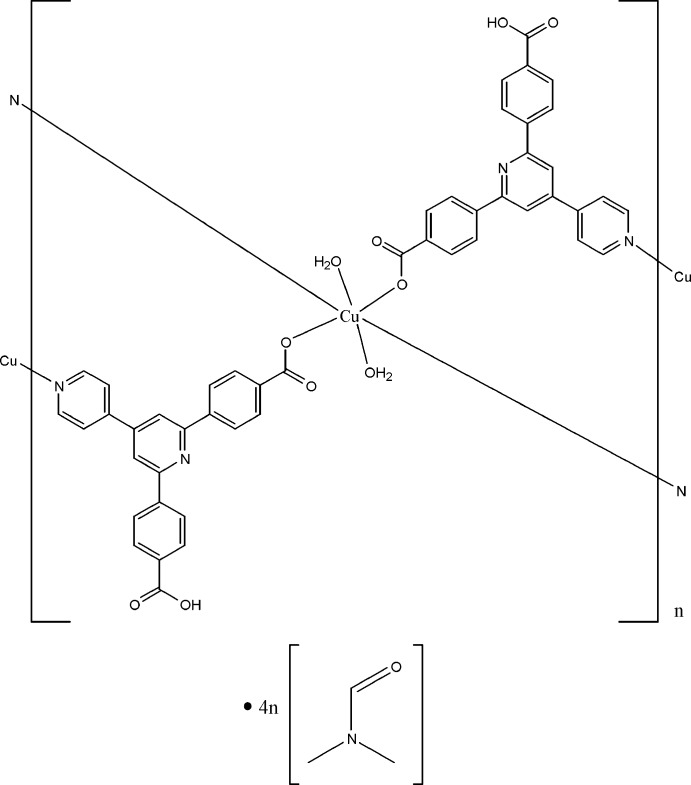


## Experimental

### 

#### Crystal data

[Cu(C_24_H_15_N_2_O_4_)_2_(H_2_O)_2_]·4C_3_H_7_NO
*M* *_r_* = 1182.73
Monoclinic, 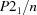

*a* = 7.7161 (17) Å
*b* = 17.550 (4) Å
*c* = 20.947 (4) Å
β = 96.800 (4)°
*V* = 2816.6 (10) Å^3^
*Z* = 2
Mo *K*α radiation
μ = 0.46 mm^−1^
*T* = 293 K
0.27 × 0.25 × 0.20 mm

#### Data collection

Bruker APEXII CCD diffractometer
Absorption correction: multi-scan (*SADABS*; Bruker, 2001 ▶) *T*_min_ = 0.885, *T*_max_ = 0.913
14622 measured reflections
5226 independent reflections
3371 reflections with *I* > 2σ(*I*)
*R*_int_ = 0.058

#### Refinement

*R*[*F* ^2^ > 2σ(*F* ^2^)] = 0.061
*wR*(*F* ^2^) = 0.185
*S* = 1.04
5226 reflections
376 parameters
H-atom parameters constrained
Δρ_max_ = 0.93 e Å^−3^
Δρ_min_ = −0.39 e Å^−3^

### 

Data collection: *APEX2* (Bruker, 2007 ▶); cell refinement: *SAINT* (Bruker, 2007 ▶); data reduction: *SAINT*; program(s) used to solve structure: *SHELXTL* (Sheldrick, 2008 ▶); program(s) used to refine structure: *SHELXTL*; molecular graphics: *XP* in *SHELXTL* and *DIAMOND* (Brandenburg, 1999 ▶); software used to prepare material for publication: *SHELXTL* (Sheldrick, 2008 ▶).

## Supplementary Material

Crystal structure: contains datablock(s) global, I. DOI: 10.1107/S1600536813006430/hy2619sup1.cif

Structure factors: contains datablock(s) I. DOI: 10.1107/S1600536813006430/hy2619Isup2.hkl

Additional supplementary materials:  crystallographic information; 3D view; checkCIF report

## Figures and Tables

**Table 1:** Hydrogen-bond geometry (Å, °)

*D*—H⋯*A*	*D*—H	H⋯*A*	*D*⋯*A*	*D*—H⋯*A*
O4—H4*A*⋯O2^i^	0.82	1.86	2.584 (4)	146
O1*W*—H1*A*⋯O5^ii^	0.85	1.98	2.808 (5)	165
O1*W*—H1*B*⋯O2^iii^	0.85	1.95	2.758 (4)	159

Symmetry codes: (i) 
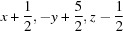
; (ii) 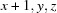; (iii) 
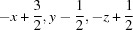
.

**Fig. 1.**
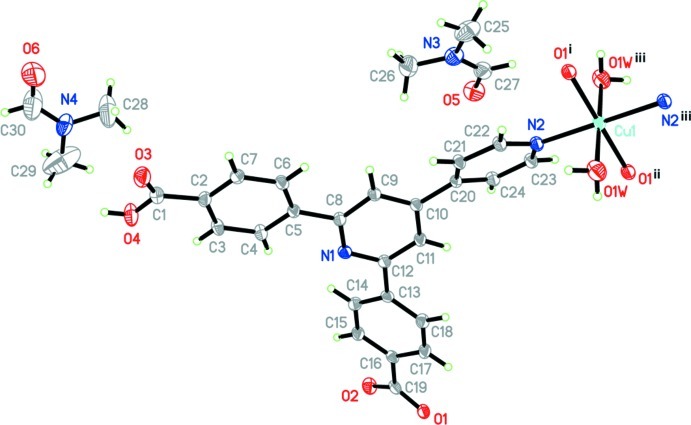
The asymmetric unit of the title compound. Displacement ellipsoids are drawn at the 30% probability level. [Symmetry codes: (i) 1/2 + x, 3/2 - y, -1/2 + z; (ii) 3/2 - x, -1/2 + y, 1/2 - z; (iii) 2 - x, 1 - y, -z.]

**Fig. 2.**
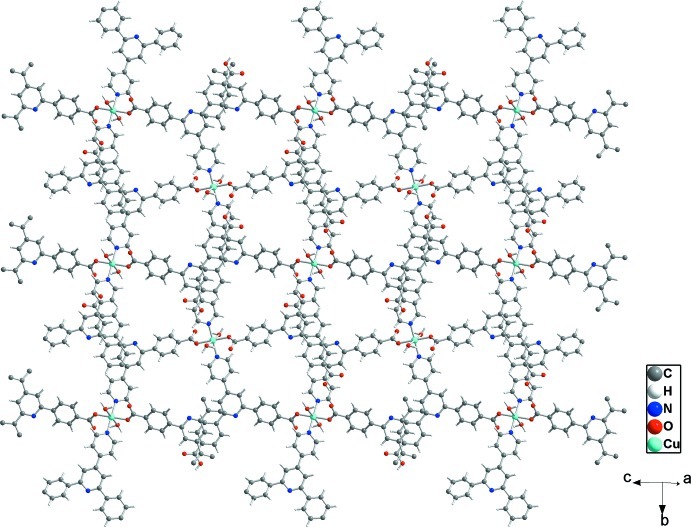
View of the layer structure of the title compound.

## supplementary crystallographic information

### Comment

Metal-organic coordination polymers (MOCPs) with infinite one-, two- or three-dimensional structures are assembled with metal ions or polynuclear clusters as nodes and organic ligands as linkers (Herm *et al.*, 2011; Liu *et al.*, 2010). Recently, the chemists have devoted themselves to design and synthesize MOCPs, not only due to their potential applications in the realm of gas adsorption and separation, catalysis, magnetism, luminescence, host–guest chemistry and *etc*, but also for their aesthetic and often complicated architectures and topologies (Ge & Song, 2012; Wang *et al.*, 2010). In order to extend the investigations in this field, we used a multifunctional ligand, 4,4'-(4,4'-bipyridine-2,6-diyl)dibenzoic acid (bpydbH_2_) to design and synthesize the title copper(II) complex and report its structure here.

The asymmetric unit of the title compound contains one Cu^II^ ion lying on an inversion center, one anionic bpydbH ligand, one aqua ligand and two lattice DMF molecules. As shown in Fig. 1, the Cu^II^ ion is six-coordinated by two N atoms from two bpydbH ligands, two deprotonated carboxylate O atoms from two other symmetry-related bpydbH ligands and two aqua ligands, furnishing a slightly distorted octahedral geometry. The bond distances and angles are in a normal range (Xia *et al.*, 2012). The Cu nodes are extended by the bridging bpydbH linkers into a layer parallel to (101) (Fig. 2). The presence of intralayer O—H···O hydrogen bonds and π–π interactions between the pyridine and benzene rings [centroid–centroid diatances = 3.808 (2) and 3.927 (2) Å] stabilizes the single layer.

### Experimental

Cu(NO_3_)_2_.3H_2_O (0.0063 g, 0.025 mmol) and bpydbH_2_ (0.0099 g, 0.025 mmol) were suspended in a mixed solvent of dimethylformamide (DMF) (4 ml) and H_2_O (0.5 ml), and heated in a 15 ml Teflon-lined stainless-steel autoclave at 80°C for 3 days. After the autoclave was cooled to room temperature slowly, green crystals were collected by filtration and washed with DMF, and dried in air (yield: 65% based on Cu).

### Refinement

H atoms on C and carboxyl O atoms were positioned geometrically and refined as riding atoms, with C—H = 0.93, 0.96 and O—H = 0.82 Å and with *U*_iso_(H) = 1.2(1.5 for methyl and carboxyl)*U*_eq_(C,*O*). H atoms of water molecules were located in a difference Fourier map and refined as riding atoms, with *U*_iso_(H) = 1.5*U*_eq_(O).

### Crystal data

**Table:**

[Cu(C_24_H_15_N_2_O_4_)_2_(H_2_O)_2_]·4C_3_H_7_NO	*F*(000) = 1238
*M**_r_* = 1182.73	*D*_x_ = 1.395 Mg m^−^^3^
Monoclinic, *P*2_1_/*n*	Mo *K*α radiation, λ = 0.71073 Å
Hall symbol: -P 2yn	Cell parameters from 5226 reflections
*a* = 7.7161 (17) Å	θ = 1.0–26.0°
*b* = 17.550 (4) Å	µ = 0.46 mm^−^^1^
*c* = 20.947 (4) Å	*T* = 293 K
β = 96.800 (4)°	Block, green
*V* = 2816.6 (10) Å^3^	0.27 × 0.25 × 0.20 mm
*Z* = 2	

### Data collection

**Table:**

Bruker APEXII CCD diffractometer	5226 independent reflections
Radiation source: fine-focus sealed tube	3371 reflections with *I* > 2σ(*I*)
Graphite monochromator	*R*_int_ = 0.058
φ and ω scans	θ_max_ = 25.5°, θ_min_ = 2.0°
Absorption correction: multi-scan (*SADABS*; Bruker, 2001)	*h* = −9→7
*T*_min_ = 0.885, *T*_max_ = 0.913	*k* = −21→21
14622 measured reflections	*l* = −20→25

### Refinement

**Table:**

Refinement on *F*^2^	Primary atom site location: structure-invariant direct methods
Least-squares matrix: full	Secondary atom site location: difference Fourier map
*R*[*F*^2^ > 2σ(*F*^2^)] = 0.061	Hydrogen site location: inferred from neighbouring sites
*wR*(*F*^2^) = 0.185	H-atom parameters constrained
*S* = 1.04	*w* = 1/[σ^2^(*F*_o_^2^) + (0.0891*P*)^2^ + 2.0095*P*] where *P* = (*F*_o_^2^ + 2*F*_c_^2^)/3
5226 reflections	(Δ/σ)_max_ < 0.001
376 parameters	Δρ_max_ = 0.93 e Å^−^^3^
0 restraints	Δρ_min_ = −0.39 e Å^−^^3^

### Special details

**Table:**

Geometry. All e.s.d.'s (except the e.s.d. in the dihedral angle between two l.s. planes) are estimated using the full covariance matrix. The cell e.s.d.'s are taken into account individually in the estimation of e.s.d.'s in distances, angles and torsion angles; correlations between e.s.d.'s in cell parameters are only used when they are defined by crystal symmetry. An approximate (isotropic) treatment of cell e.s.d.'s is used for estimating e.s.d.'s involving l.s. planes.
Refinement. Refinement of *F*^2^ against ALL reflections. The weighted *R*-factor *wR* and goodness of fit *S* are based on *F*^2^, conventional *R*-factors *R* are based on *F*, with *F* set to zero for negative *F*^2^. The threshold expression of *F*^2^ > σ(*F*^2^) is used only for calculating *R*-factors(gt) *etc*. and is not relevant to the choice of reflections for refinement. *R*-factors based on *F*^2^ are statistically about twice as large as those based on *F*, and *R*- factors based on ALL data will be even larger.

### Fractional atomic coordinates and isotropic or equivalent isotropic displacement parameters (Å^2^)

**Table:**

	*x*	*y*	*z*	*U*_iso_*/*U*_eq_	
Cu1	1.0000	0.5000	0.0000	0.0362 (2)	
C1	0.8452 (6)	1.2336 (2)	−0.1386 (2)	0.0432 (10)	
C2	0.8060 (5)	1.1653 (2)	−0.10151 (19)	0.0347 (9)	
C3	0.7251 (5)	1.1715 (2)	−0.0455 (2)	0.0369 (10)	
H3	0.6854	1.2186	−0.0330	0.044*	
C4	0.7040 (5)	1.1079 (2)	−0.0088 (2)	0.0350 (9)	
H4	0.6517	1.1128	0.0288	0.042*	
C5	0.7592 (5)	1.0363 (2)	−0.02669 (18)	0.0286 (8)	
C6	0.8380 (5)	1.0305 (2)	−0.08253 (19)	0.0352 (9)	
H6	0.8762	0.9832	−0.0953	0.042*	
C7	0.8607 (5)	1.0943 (2)	−0.11976 (19)	0.0376 (10)	
H7	0.9132	1.0894	−0.1573	0.045*	
C8	0.7389 (5)	0.9695 (2)	0.01565 (19)	0.0302 (8)	
C9	0.7683 (5)	0.8950 (2)	−0.00365 (18)	0.0304 (9)	
H9	0.7972	0.8853	−0.0447	0.036*	
C10	0.7540 (5)	0.83558 (19)	0.03927 (18)	0.0280 (8)	
C11	0.7072 (5)	0.8537 (2)	0.09959 (18)	0.0314 (9)	
H11	0.6958	0.8154	0.1295	0.038*	
C12	0.6774 (5)	0.9291 (2)	0.11507 (18)	0.0299 (8)	
C13	0.6253 (5)	0.9511 (2)	0.17864 (18)	0.0301 (9)	
C14	0.5242 (5)	1.0156 (2)	0.18392 (19)	0.0358 (9)	
H14	0.4924	1.0455	0.1478	0.043*	
C15	0.4702 (5)	1.0358 (2)	0.24194 (19)	0.0372 (10)	
H15	0.4004	1.0786	0.2445	0.045*	
C16	0.5196 (5)	0.9925 (2)	0.29668 (18)	0.0312 (9)	
C17	0.6242 (5)	0.9290 (2)	0.29215 (19)	0.0378 (10)	
H17	0.6586	0.8998	0.3285	0.045*	
C18	0.6777 (5)	0.9089 (2)	0.23352 (19)	0.0366 (9)	
H18	0.7494	0.8667	0.2310	0.044*	
C19	0.4600 (6)	1.0145 (2)	0.3597 (2)	0.0361 (10)	
C20	0.7944 (5)	0.75539 (19)	0.02344 (17)	0.0288 (8)	
C21	0.9191 (5)	0.73870 (19)	−0.01677 (18)	0.0307 (9)	
H21	0.9689	0.7778	−0.0384	0.037*	
C22	0.9695 (5)	0.6647 (2)	−0.02478 (19)	0.0339 (9)	
H22	1.0543	0.6549	−0.0517	0.041*	
C23	0.7732 (5)	0.6214 (2)	0.04100 (19)	0.0360 (9)	
H23	0.7197	0.5811	0.0598	0.043*	
C24	0.7177 (5)	0.6942 (2)	0.05158 (18)	0.0342 (9)	
H24	0.6294	0.7025	0.0774	0.041*	
C25	0.6692 (8)	0.6406 (4)	−0.1720 (3)	0.0829 (19)	
H25A	0.7059	0.6773	−0.2016	0.124*	
H25B	0.6144	0.5984	−0.1955	0.124*	
H25C	0.7689	0.6229	−0.1442	0.124*	
C26	0.5118 (8)	0.7558 (3)	−0.1443 (3)	0.0756 (16)	
H26A	0.5739	0.7741	−0.1783	0.113*	
H26B	0.5493	0.7833	−0.1055	0.113*	
H26C	0.3889	0.7633	−0.1558	0.113*	
C27	0.4770 (6)	0.6364 (3)	−0.0897 (2)	0.0543 (12)	
H27	0.5082	0.5854	−0.0843	0.065*	
C28	0.4517 (16)	1.2769 (5)	−0.2557 (5)	0.203 (6)	
H28A	0.4153	1.2642	−0.2148	0.304*	
H28B	0.5656	1.2561	−0.2586	0.304*	
H28C	0.3703	1.2562	−0.2895	0.304*	
C29	0.4063 (11)	1.3970 (6)	−0.2090 (4)	0.157 (4)	
H29A	0.3787	1.3613	−0.1770	0.235*	
H29B	0.3054	1.4274	−0.2228	0.235*	
H29C	0.4999	1.4294	−0.1912	0.235*	
C30	0.5028 (11)	1.3864 (4)	−0.3123 (4)	0.119 (3)	
H30	0.4860	1.4387	−0.3166	0.142*	
N1	0.6925 (4)	0.98644 (16)	0.07362 (15)	0.0305 (7)	
N2	0.9010 (4)	0.60580 (17)	0.00477 (15)	0.0331 (8)	
N3	0.5471 (5)	0.6755 (2)	−0.13415 (19)	0.0533 (10)	
N4	0.4576 (6)	1.3570 (2)	−0.2622 (2)	0.0619 (11)	
O1	0.5313 (4)	0.98064 (14)	0.40897 (13)	0.0395 (7)	
O2	0.3418 (4)	1.06386 (16)	0.35908 (14)	0.0488 (8)	
O3	0.9397 (5)	1.23445 (17)	−0.18056 (16)	0.0582 (9)	
O4	0.7673 (4)	1.29571 (17)	−0.11906 (16)	0.0607 (9)	
H4A	0.7916	1.3324	−0.1405	0.091*	
O5	0.3740 (4)	0.6624 (2)	−0.05498 (17)	0.0651 (10)	
O6	0.5670 (7)	1.3545 (2)	−0.3567 (2)	0.1082 (17)	
O1W	1.3019 (4)	0.54357 (17)	0.02770 (15)	0.0552 (8)	
H1A	1.3236	0.5847	0.0087	0.083*	
H1B	1.2828	0.5552	0.0656	0.083*	

### Atomic displacement parameters (Å^2^)

**Table:**

	*U*^11^	*U*^22^	*U*^33^	*U*^12^	*U*^13^	*U*^23^
Cu1	0.0628 (5)	0.0222 (3)	0.0263 (4)	0.0050 (3)	0.0164 (3)	0.0015 (3)
C1	0.054 (3)	0.033 (2)	0.042 (3)	−0.001 (2)	0.005 (2)	0.0034 (19)
C2	0.036 (2)	0.031 (2)	0.037 (2)	−0.0049 (17)	0.0019 (18)	0.0061 (17)
C3	0.040 (2)	0.0251 (19)	0.046 (3)	0.0037 (16)	0.010 (2)	0.0004 (17)
C4	0.039 (2)	0.0273 (19)	0.041 (2)	0.0008 (17)	0.0146 (19)	0.0013 (17)
C5	0.032 (2)	0.0272 (19)	0.027 (2)	−0.0010 (16)	0.0060 (16)	0.0009 (15)
C6	0.048 (2)	0.0254 (18)	0.033 (2)	0.0009 (17)	0.0094 (19)	−0.0029 (16)
C7	0.052 (3)	0.035 (2)	0.028 (2)	−0.0037 (18)	0.0104 (19)	0.0002 (17)
C8	0.034 (2)	0.0256 (18)	0.032 (2)	0.0004 (16)	0.0066 (17)	0.0011 (16)
C9	0.037 (2)	0.0284 (19)	0.027 (2)	0.0006 (16)	0.0088 (17)	−0.0017 (16)
C10	0.033 (2)	0.0240 (18)	0.028 (2)	−0.0001 (15)	0.0067 (16)	−0.0014 (15)
C11	0.042 (2)	0.0257 (19)	0.027 (2)	0.0008 (16)	0.0090 (17)	0.0019 (16)
C12	0.036 (2)	0.0275 (19)	0.027 (2)	−0.0004 (16)	0.0085 (17)	0.0000 (16)
C13	0.038 (2)	0.0284 (19)	0.025 (2)	−0.0028 (16)	0.0074 (17)	−0.0047 (15)
C14	0.048 (2)	0.033 (2)	0.027 (2)	0.0025 (17)	0.0097 (19)	0.0007 (16)
C15	0.050 (2)	0.030 (2)	0.034 (2)	0.0050 (18)	0.0104 (19)	−0.0020 (17)
C16	0.041 (2)	0.0259 (19)	0.029 (2)	−0.0039 (16)	0.0126 (17)	−0.0045 (16)
C17	0.051 (3)	0.032 (2)	0.032 (2)	0.0009 (18)	0.0092 (19)	0.0052 (17)
C18	0.047 (2)	0.031 (2)	0.033 (2)	0.0078 (18)	0.0118 (19)	−0.0013 (17)
C19	0.050 (2)	0.026 (2)	0.035 (2)	−0.0054 (18)	0.014 (2)	−0.0043 (17)
C20	0.039 (2)	0.0239 (18)	0.024 (2)	0.0012 (16)	0.0051 (16)	−0.0003 (15)
C21	0.043 (2)	0.0225 (18)	0.029 (2)	−0.0019 (16)	0.0141 (17)	0.0005 (15)
C22	0.047 (2)	0.0275 (19)	0.030 (2)	−0.0006 (17)	0.0146 (18)	−0.0004 (16)
C23	0.052 (3)	0.028 (2)	0.030 (2)	−0.0039 (18)	0.0140 (19)	0.0014 (16)
C24	0.045 (2)	0.031 (2)	0.029 (2)	0.0012 (17)	0.0158 (18)	−0.0017 (16)
C25	0.081 (4)	0.119 (5)	0.052 (4)	0.025 (4)	0.022 (3)	0.004 (3)
C26	0.097 (4)	0.062 (3)	0.069 (4)	−0.003 (3)	0.017 (3)	0.009 (3)
C27	0.056 (3)	0.053 (3)	0.054 (3)	−0.005 (2)	0.005 (3)	−0.002 (2)
C28	0.303 (15)	0.088 (6)	0.196 (11)	−0.064 (8)	−0.057 (10)	0.066 (7)
C29	0.132 (7)	0.259 (12)	0.082 (6)	0.104 (8)	0.025 (5)	0.000 (6)
C30	0.167 (8)	0.079 (5)	0.123 (7)	0.022 (5)	0.067 (6)	0.031 (5)
N1	0.0353 (17)	0.0303 (17)	0.0272 (18)	−0.0014 (13)	0.0090 (14)	−0.0030 (13)
N2	0.049 (2)	0.0260 (16)	0.0265 (18)	0.0009 (14)	0.0128 (15)	0.0000 (13)
N3	0.054 (2)	0.062 (3)	0.046 (2)	0.000 (2)	0.0151 (19)	0.0015 (19)
N4	0.071 (3)	0.059 (3)	0.061 (3)	0.012 (2)	0.027 (2)	0.019 (2)
O1	0.0655 (19)	0.0274 (14)	0.0276 (16)	−0.0003 (13)	0.0135 (14)	−0.0001 (11)
O2	0.071 (2)	0.0377 (16)	0.0413 (19)	0.0105 (15)	0.0232 (15)	−0.0045 (13)
O3	0.082 (2)	0.0458 (19)	0.051 (2)	−0.0038 (17)	0.0217 (19)	0.0064 (15)
O4	0.081 (2)	0.0349 (17)	0.070 (2)	0.0016 (16)	0.0251 (19)	0.0134 (16)
O5	0.060 (2)	0.075 (2)	0.064 (2)	−0.0093 (18)	0.0235 (19)	−0.0012 (19)
O6	0.179 (5)	0.077 (3)	0.083 (3)	−0.005 (3)	0.075 (3)	−0.021 (2)
O1W	0.072 (2)	0.0441 (18)	0.053 (2)	−0.0041 (16)	0.0239 (17)	−0.0006 (15)

### Geometric parameters (Å, º)

**Table:**

Cu1—O1^i^	1.980 (3)	C19—O2	1.256 (5)
Cu1—O1^ii^	1.980 (3)	C19—O1	1.260 (5)
Cu1—N2	2.015 (3)	C20—C21	1.383 (5)
Cu1—N2^iii^	2.015 (3)	C20—C24	1.390 (5)
C1—O3	1.207 (5)	C21—C22	1.371 (5)
C1—O4	1.332 (5)	C21—H21	0.9300
C1—C2	1.480 (5)	C22—N2	1.345 (5)
C2—C7	1.383 (5)	C22—H22	0.9300
C2—C3	1.398 (5)	C23—N2	1.342 (5)
C3—C4	1.375 (5)	C23—C24	1.374 (5)
C3—H3	0.9300	C23—H23	0.9300
C4—C5	1.393 (5)	C24—H24	0.9300
C4—H4	0.9300	C25—N3	1.439 (6)
C5—C6	1.385 (5)	C25—H25A	0.9600
C5—C8	1.490 (5)	C25—H25B	0.9600
C6—C7	1.388 (5)	C25—H25C	0.9600
C6—H6	0.9300	C26—N3	1.447 (6)
C7—H7	0.9300	C26—H26A	0.9600
C8—N1	1.340 (5)	C26—H26B	0.9600
C8—C9	1.394 (5)	C26—H26C	0.9600
C9—C10	1.390 (5)	C27—O5	1.226 (5)
C9—H9	0.9300	C27—N3	1.323 (6)
C10—C11	1.392 (5)	C27—H27	0.9300
C10—C20	1.487 (5)	C28—N4	1.413 (9)
C11—C12	1.388 (5)	C28—H28A	0.9600
C11—H11	0.9300	C28—H28B	0.9600
C12—N1	1.343 (5)	C28—H28C	0.9600
C12—C13	1.487 (5)	C29—N4	1.411 (8)
C13—C14	1.385 (5)	C29—H29A	0.9600
C13—C18	1.387 (5)	C29—H29B	0.9600
C14—C15	1.377 (5)	C29—H29C	0.9600
C14—H14	0.9300	C30—O6	1.238 (8)
C15—C16	1.391 (5)	C30—N4	1.255 (7)
C15—H15	0.9300	C30—H30	0.9300
C16—C17	1.385 (5)	O1—Cu1^iv^	1.980 (3)
C16—C19	1.499 (5)	O4—H4A	0.8200
C17—C18	1.387 (5)	O1W—H1A	0.8501
C17—H17	0.9300	O1W—H1B	0.8489
C18—H18	0.9300		
			
O1^i^—Cu1—O1^ii^	180.0	O2—C19—C16	117.9 (4)
O1^i^—Cu1—N2	91.16 (11)	O1—C19—C16	116.7 (4)
O1^ii^—Cu1—N2	88.84 (11)	C21—C20—C24	117.2 (3)
O1^i^—Cu1—N2^iii^	88.84 (11)	C21—C20—C10	121.0 (3)
O1^ii^—Cu1—N2^iii^	91.16 (11)	C24—C20—C10	121.7 (3)
N2—Cu1—N2^iii^	180.0	C22—C21—C20	120.2 (3)
O3—C1—O4	123.2 (4)	C22—C21—H21	119.9
O3—C1—C2	124.8 (4)	C20—C21—H21	119.9
O4—C1—C2	112.0 (4)	N2—C22—C21	122.6 (4)
C7—C2—C3	118.9 (3)	N2—C22—H22	118.7
C7—C2—C1	119.8 (4)	C21—C22—H22	118.7
C3—C2—C1	121.1 (4)	N2—C23—C24	123.0 (3)
C4—C3—C2	120.0 (4)	N2—C23—H23	118.5
C4—C3—H3	120.0	C24—C23—H23	118.5
C2—C3—H3	120.0	C23—C24—C20	119.6 (4)
C3—C4—C5	121.4 (4)	C23—C24—H24	120.2
C3—C4—H4	119.3	C20—C24—H24	120.2
C5—C4—H4	119.3	N3—C25—H25A	109.5
C6—C5—C4	118.3 (3)	N3—C25—H25B	109.5
C6—C5—C8	122.2 (3)	H25A—C25—H25B	109.5
C4—C5—C8	119.5 (3)	N3—C25—H25C	109.5
C5—C6—C7	120.8 (4)	H25A—C25—H25C	109.5
C5—C6—H6	119.6	H25B—C25—H25C	109.5
C7—C6—H6	119.6	N3—C26—H26A	109.5
C2—C7—C6	120.6 (4)	N3—C26—H26B	109.5
C2—C7—H7	119.7	H26A—C26—H26B	109.5
C6—C7—H7	119.7	N3—C26—H26C	109.5
N1—C8—C9	122.8 (3)	H26A—C26—H26C	109.5
N1—C8—C5	115.0 (3)	H26B—C26—H26C	109.5
C9—C8—C5	122.2 (3)	O5—C27—N3	124.9 (5)
C10—C9—C8	119.2 (3)	O5—C27—H27	117.6
C10—C9—H9	120.4	N3—C27—H27	117.6
C8—C9—H9	120.4	N4—C28—H28A	109.5
C9—C10—C11	117.7 (3)	N4—C28—H28B	109.5
C9—C10—C20	122.1 (3)	H28A—C28—H28B	109.5
C11—C10—C20	120.2 (3)	N4—C28—H28C	109.5
C12—C11—C10	119.9 (3)	H28A—C28—H28C	109.5
C12—C11—H11	120.1	H28B—C28—H28C	109.5
C10—C11—H11	120.1	N4—C29—H29A	109.5
N1—C12—C11	122.3 (3)	N4—C29—H29B	109.5
N1—C12—C13	115.9 (3)	H29A—C29—H29B	109.5
C11—C12—C13	121.8 (3)	N4—C29—H29C	109.5
C14—C13—C18	118.8 (3)	H29A—C29—H29C	109.5
C14—C13—C12	119.9 (3)	H29B—C29—H29C	109.5
C18—C13—C12	121.3 (3)	O6—C30—N4	128.2 (7)
C15—C14—C13	120.9 (4)	O6—C30—H30	115.9
C15—C14—H14	119.6	N4—C30—H30	115.9
C13—C14—H14	119.6	C8—N1—C12	118.2 (3)
C14—C15—C16	120.3 (4)	C23—N2—C22	117.3 (3)
C14—C15—H15	119.9	C23—N2—Cu1	121.6 (2)
C16—C15—H15	119.9	C22—N2—Cu1	120.9 (3)
C17—C16—C15	119.2 (4)	C27—N3—C25	121.1 (5)
C17—C16—C19	120.7 (4)	C27—N3—C26	121.6 (4)
C15—C16—C19	120.1 (3)	C25—N3—C26	117.3 (4)
C16—C17—C18	120.2 (4)	C30—N4—C29	125.9 (7)
C16—C17—H17	119.9	C30—N4—C28	120.2 (7)
C18—C17—H17	119.9	C29—N4—C28	113.8 (7)
C17—C18—C13	120.6 (4)	C19—O1—Cu1^iv^	128.1 (3)
C17—C18—H18	119.7	C1—O4—H4A	109.5
C13—C18—H18	119.7	H1A—O1W—H1B	107.4
O2—C19—O1	125.4 (4)		
			
O3—C1—C2—C7	−8.6 (7)	C19—C16—C17—C18	179.6 (4)
O4—C1—C2—C7	173.1 (4)	C16—C17—C18—C13	−1.0 (6)
O3—C1—C2—C3	166.8 (4)	C14—C13—C18—C17	2.5 (6)
O4—C1—C2—C3	−11.4 (6)	C12—C13—C18—C17	−178.2 (3)
C7—C2—C3—C4	1.3 (6)	C17—C16—C19—O2	−167.7 (4)
C1—C2—C3—C4	−174.2 (4)	C15—C16—C19—O2	12.3 (5)
C2—C3—C4—C5	−1.1 (6)	C17—C16—C19—O1	11.0 (5)
C3—C4—C5—C6	0.6 (6)	C15—C16—C19—O1	−168.9 (4)
C3—C4—C5—C8	178.0 (4)	C9—C10—C20—C21	30.9 (6)
C4—C5—C6—C7	−0.2 (6)	C11—C10—C20—C21	−146.3 (4)
C8—C5—C6—C7	−177.6 (4)	C9—C10—C20—C24	−153.5 (4)
C3—C2—C7—C6	−1.0 (6)	C11—C10—C20—C24	29.2 (5)
C1—C2—C7—C6	174.6 (4)	C24—C20—C21—C22	−3.5 (6)
C5—C6—C7—C2	0.5 (6)	C10—C20—C21—C22	172.2 (4)
C6—C5—C8—N1	167.3 (3)	C20—C21—C22—N2	0.6 (6)
C4—C5—C8—N1	−10.0 (5)	N2—C23—C24—C20	0.7 (6)
C6—C5—C8—C9	−11.9 (6)	C21—C20—C24—C23	2.9 (6)
C4—C5—C8—C9	170.7 (4)	C10—C20—C24—C23	−172.8 (4)
N1—C8—C9—C10	−1.8 (6)	C9—C8—N1—C12	1.4 (5)
C5—C8—C9—C10	177.3 (3)	C5—C8—N1—C12	−177.9 (3)
C8—C9—C10—C11	1.2 (5)	C11—C12—N1—C8	−0.4 (5)
C8—C9—C10—C20	−176.1 (3)	C13—C12—N1—C8	−179.9 (3)
C9—C10—C11—C12	−0.3 (5)	C24—C23—N2—C22	−3.6 (6)
C20—C10—C11—C12	177.1 (3)	C24—C23—N2—Cu1	171.8 (3)
C10—C11—C12—N1	−0.1 (6)	C21—C22—N2—C23	3.0 (6)
C10—C11—C12—C13	179.4 (3)	C21—C22—N2—Cu1	−172.4 (3)
N1—C12—C13—C14	28.6 (5)	O1^i^—Cu1—N2—C23	−34.5 (3)
C11—C12—C13—C14	−151.0 (4)	O1^ii^—Cu1—N2—C23	145.5 (3)
N1—C12—C13—C18	−150.7 (4)	O1^i^—Cu1—N2—C22	140.8 (3)
C11—C12—C13—C18	29.8 (6)	O1^ii^—Cu1—N2—C22	−39.2 (3)
C18—C13—C14—C15	−2.7 (6)	O5—C27—N3—C25	179.0 (5)
C12—C13—C14—C15	178.1 (4)	O5—C27—N3—C26	2.5 (8)
C13—C14—C15—C16	1.3 (6)	O6—C30—N4—C29	171.2 (8)
C14—C15—C16—C17	0.3 (6)	O6—C30—N4—C28	−10.0 (14)
C14—C15—C16—C19	−179.8 (4)	O2—C19—O1—Cu1^iv^	−6.5 (6)
C15—C16—C17—C18	−0.4 (6)	C16—C19—O1—Cu1^iv^	174.9 (2)

Symmetry codes: (i) −*x*+3/2, *y*−1/2, −*z*+1/2; (ii) *x*+1/2, −*y*+3/2, *z*−1/2; (iii) −*x*+2, −*y*+1, −*z*; (iv) −*x*+3/2, *y*+1/2, −*z*+1/2.

### Hydrogen-bond geometry (Å, º)

**Table:**

*D*—H···*A*	*D*—H	H···*A*	*D*···*A*	*D*—H···*A*
O4—H4*A*···O2^v^	0.82	1.86	2.584 (4)	146
O1*W*—H1*A*···O5^vi^	0.85	1.98	2.808 (5)	165
O1*W*—H1*B*···O2^i^	0.85	1.95	2.758 (4)	159

Symmetry codes: (i) −*x*+3/2, *y*−1/2, −*z*+1/2; (v) *x*+1/2, −*y*+5/2, *z*−1/2; (vi) *x*+1, *y*, *z*.
